# Neuronal Plasticity-Dependent Paradigm and Young Plasma Treatment Prevent Synaptic and Motor Deficit in a Rett Syndrome Mouse Model

**DOI:** 10.3390/biom15050748

**Published:** 2025-05-21

**Authors:** Sofía Espinoza, Camila Navia, Rodrigo F. Torres, Nuria Llontop, Verónica Valladares, Cristina Silva, Ariel Vivero, Exequiel Novoa-Padilla, Jessica Soto-Covasich, Jessica Mella, Ricardo Kouro, Sharin Valdivia, Marco Pérez-Bustamante, Patricia Ojeda-Provoste, Nancy Pineda, Sonja Buvinic, Dasfne Lee-Liu, Juan Pablo Henríquez, Bredford Kerr

**Affiliations:** 1 Centro de Biología Celular y Biomedicina (CEBICEM), Facultad de Medicina y Ciencia, Universidad San Sebastián, Providencia, Santiago 7510157, Chilenllontopl@correo.uss.cl (N.L.); avive@inta.uchile.cl (A.V.); 2 Centro de Estudios Científicos (CECs), Valdivia 5110466, Chilericardo.kouro@alumnos.uach.cl (R.K.);; 3 Facultad de Medicina, Universidad Austral de Chile, Valdivia 5091000, Chile; 4 Departamento de Ciencias Básicas, Facultad de Medicina y Ciencia, Universidad San Sebastián, Puerto Montt 5501842, Chile; 5 Neuromuscular Studies Laboratory (NeSt Lab), Instituto de Anatomía, Histología y Patología, Facultad de Medicina, Universidad Austral de Chile, Valdivia 5091000, Chile; jessica.mella@uchile.cl (J.M.);; 6 Facultad de Ciencias Biológicas, Universidad de Concepción, Concepción 4070386, Chile; 7 Facultad de Ciencias, Universidad Austral de Chile, Valdivia 5091000, Chile; 8 Departamento de Ciencias Biológicas y Químicas, Facultad de Ciencias, Universidad San Sebastián, Concepción 4080870, Chile; 9 Facultad de Odontología, Universidad de Chile, Santiago 8380000, Chile; sbuvinic@u.uchile.cl; 10 Escuela de Química y Farmacia, Facultad de Medicina y Ciencia, Universidad San Sebastián, Santiago 7510157, Chile

**Keywords:** Rett syndrome, *Mecp2*, environmental enrichment, plasma treatment

## Abstract

Classical Rett syndrome (RTT) is a neurodevelopmental disorder caused by mutations in the *MECP2* gene, resulting in a devastating phenotype associated with a lack of gene expression control. Mouse models lacking *Mecp2* expression with an RTT-like phenotype have been developed to advance therapeutic alternatives. Environmental enrichment (EE) attenuates RTT symptoms in patients and mouse models. However, the mechanisms underlying the effects of EE on RTT have not been fully elucidated. We housed male hemizygous *Mecp2*-null (*Mecp2^-/y^*) and wild-type mice in specially conditioned cages to enhance sensory, cognitive, social, and motor stimulation. EE attenuated the progression of the RTT phenotype by preserving neuronal cytoarchitecture and neural plasticity markers. Furthermore, EE ameliorated defects in neuromuscular junction organization and restored the motor deficit of *Mecp2^-/y^* mice. Treatment with plasma from young WT mice was used to assess whether the increased activity could modify plasma components, mimicking the benefits of EE in *Mecp2^-/y^*. Plasma treatment attenuated the RTT phenotype by improving neurological markers, suggesting that peripheral signals of mice with normal motor function have the potential to reactivate dormant neurodevelopment in RTT mice. These findings demonstrate how EE and treatment with young plasma ameliorate RTT-like phenotype in mice, opening new therapeutical approaches for RTT patients.

## 1. Introduction

Classical Rett syndrome (RTT, OMIM #312750) is a devastating neurodevelopmental disorder and one of the leading causes of cognitive deficit in young women, with an incidence of 1 in 5000–10,000 female births [1,2], and a prevalence of 5 to 10 per 100,000 females [3]. Rett syndrome patients have a seemingly normal neurological development during the first 6–18 months of life, followed by stagnation, and later fall into a developmental regression accompanied by the onset of symptoms such as motor impairment, loss of hand skills, seizure, autonomic dysfunction, anxiety alterations, and intellectual disabilities, among other neurological manifestations [4,5,6]. In addition to these nervous system-associated phenotypes, RTT patients exhibit peripheral alterations such as respiratory abnormalities [7] and evidence of axonopathy with skeletal muscle alterations as a probable consequence of denervation [8]. Cardiorespiratory failure has been reported as one of the most prevalent causes of death in RTT patients [9]. 

Mutations in the X-linked gene *MECP2* are the leading cause of RTT [4,10,11]. *MECP2* encodes two isoforms of the Methyl CpG Binding Protein-2 (MECP2). This transcriptional regulator binds to methylated and hydroxymethylated cytosine in CpG dinucleotides to recruit transcriptional regulatory complexes to either decrease or increase the expression of its target genes [12,13]. At the time of birth, MECP2 expression is low, but its expression gradually increases until it reaches the highest levels in mature neurons [14,15]. This explains the RTT phenotype as neurodevelopment progresses and highlights the role of MECP2 as a DNA methylation/hydroxymethylation reader critical to maintaining neuronal functions in the mature brain.

Due to the monogenetic nature of Rett syndrome, several mouse models lacking *Mecp2* expression have been developed to investigate the pathophysiological and cellular bases of RTT and uncover the molecular mechanisms underlying the RTT phenotype [16,17,18]. These mouse models recapitulate most but not all the RTT phenotype observed in RTT patients and have been extensively characterized [16,17,19,20,21]. Studies using RTT mouse models have shown that neurons lacking the expression of *Mecp2* postnatally exhibit altered maintenance of the mature neuronal network [22]. In addition, conditional re-expression of Mecp2 in adult hemizygous *Mecp2*-null mutant (*Mecp2^-/y^*) mice rescues most RTT phenotypes [23,24,25,26]. This evidence demonstrates that Mecp2 is required to maintain neuronal function and that its absence does not irreversibly affect the arrested neurodevelopment observed in RTT. This evidence provides an opportunity for developing potential therapeutic interventions for RTT patients.

Evidence shows that RTT-associated phenotype may result from an alteration in dendritic complexity and spine dysgenesis [27]. Indeed, postmortem fixed brains from RTT patients exhibit reduced dendritic complexity and decreased spine density [4,28,29,30]. Moreover, iPSC-derived neurons from RTT patients exhibit defects in neuronal maturation and synaptic formation [31,32]. As noted, RTT-mouse models offer a platform for studying brain alterations associated with RTT, as they replicate most of the phenotypes in RTT patients. For instance, the decreased synaptic parameters observed in RTT patients have also been observed in layer II/III of the motor cortex and layer V of the somatosensory cortex of *Mecp2^-/y^* mice [33,34,35,36]. In addition, the number of excitatory synapses observed in primary cortical cultures of *Mecp2*-knockdown mice is reduced compared to wild-type mice [37]. These findings support the hypothesis that impaired synaptic maturation underlies the neuronal dysfunction observed in RTT. Thus, interventions to prevent or reverse neural decline may represent promising therapeutic approaches for RTT patients.

Environmental enrichment (EE) is an experimental paradigm widely used in mouse models to enhance neuronal plasticity and modulate the pathogenesis of central nervous system disorders through molecular, cellular, and behavioral effects [38]. This experimental paradigm has shown promising results in RTT mouse models and has even been proposed as a therapeutic alternative not only for RTT patients [39] but also for other neurodevelopmental disorders [40]. This hypothesis is supported by evidence from independent groups using different RTT mouse models. Kondo et al. demonstrated that EE ameliorates the motor coordination deficits of heterozygous *Mecp2*-null females exposed to EE for 4 weeks; however, this paradigm was ineffective in *Mecp2^-/y^* males [41]. Subsequently, it was demonstrated that EE attenuates locomotor deficit in *Mecp2^-/y^* male mice exposed to EE since weaning, likely by increasing brain matter [42] and by inducing an unconventional transcriptional response that is not associated with enhanced expression of synaptic markers in mice exposed temporarily to an EE housing [43]. These studies underscore the importance of an early intervention to attenuate the progression of RTT-like symptoms. Moreover, presymptomatic training dramatically improves the performance of specific motor and memory tasks, delaying the onset of symptoms [44]. Lonetti et al. demonstrated that EE promotes synaptic plasticity and synapse formation in *Mecp2^-/y^* male mice exposed to EE from 10 days of age before RTT-related phenotypes are evident [45]. *Mecp2^-/y^* mice exhibit decreased locomotor activity and neuronal plasticity [43,46], and EE includes elements that encourage increased locomotor activity, which has been described to enhance circulating levels of neurotrophins, potentially impacting the central nervous system [47,48]. Moreover, treatment with young plasma effectively rescues the attenuated neural plasticity observed in aged mice [49]. These findings suggest that elements in the plasma of mice with regular motor activity might reflect changes in the plasma of *Mecp2^-/y^* mice exposed to EE.

We aimed to gain insight into the mechanism by which EE promotes brain gain of function and attenuates the RTT-like phenotype in a mouse model of the disease with the goal of designing plausible intervention strategies for patients. To this end, we housed *Mecp2^-/y^* and wild-type (WT) male mice in specially conditioned cages to enhance sensory, cognitive, social, and motor stimulation. We compared behavioral, molecular, and cellular parameters between *Mecp2^-/y^* mice exposed to EE and those housed in regular conditions. We found that continuous exposure to EE attenuates the progression of the RTT phenotype by preventing damage to neuronal cytoarchitecture. These effects were partially replicated by intraperitoneal plasma injection from healthy young mice. These results show that early and permanent exposure to EE attenuates the RTT-phenotype progression by a mechanism associated with preventing cytoarchitecture deterioration. Additionally, this EE-induced effect was partially emulated by treatment with plasma from WT young mice, indicating that peripheral signals present in mice with regular motor activity prevent brain cytoarchitecture deterioration and attenuate the RTT progression. 

## 2. Materials and Methods

### 2.1. Mice, Housing Conditions, and Genotyping

To determine the effect of EE exposure on RTT-like phenotype, we used the *Mecp2^-/y^* mouse line generated by Adrian Bird’s lab [16]. Considering the fertility and maternal care provided by females in the 129/SvJ genetic background, heterozygous *Mecp2*-null females from a C57BL/6 genetic background (obtained from Jackson Laboratory stock #003890) were bred with 129/SvJ wild-type males for at least 10 generations. We then generated *Mecp2^-/y^* male mice in a mixed homogeneous C57BL/6:129SvJ genetic background obtained by mating heterozygous *Mecp2*-null 129SvJ females with WT C57BL/6 males. 

The genotype of the mice was determined by PCR analysis of DNA extracted from a tail biopsy at 14-21 days of age to identify transgenic mice using the following primers: F: CCACCCTCCAGTTTGGTTTA, R1: GACCCCTTGGGACTGAAGTT, and R2: CCATGCGATAAGCTTGATGA. At weaning, *Mecp2^-/y^* mice and their WT littermates were randomly housed in either standard conditions (SC) or environmental enrichment cages (EE) until being evaluated. SC comprises 3–4 mice housed in a 30 × 15 cm cage provided with bedding and ad libitum access to food (SD, Envigo S2019 and LabDiet 5P00 Prolab-RMH-3000) and water, whereas EE cages consisted of two connected 30 × 30 cm cages housing 7–8 mice with access to different bedding material, daily-changed plastic toys, a free-running wheel, and ad libitum access to water and food contained in various containers and located in different places. Both types of cages were kept in the same room and individually ventilated. All protocols were designed according to the Guide for the Care and Use of Laboratory Animals published by the US National Institutes of Health (NIH publication no. 85-23, revised 2011) and approved by the Centro de Estudios Científicos Animal Care and Use Committee, protocol number AAF#CECs 2011-03 and AAF#CECs 2011-04.

### 2.2. Overall Phenotype and Behavioral Tests

To evaluate the RTT-like phenotype progression, mice in both SC or EE were assessed weekly in their overall state starting from 3 weeks of age by measuring body weight, lifespan, hindlimb clasping, tremor, and coat condition, as previously described [43,46,50]. Additionally, the presence of ataxia in the gait as a consequence of cerebellar dysfunction in motor coordination was evaluated through a ledge test. The level of RTT-phenotype severity was determined according to an arbitrary scaling from 0 to 3, in which 0 means the absence of the phenotype and 3 is a severe phenotype [46]. Clasping: 0, no clasping; 1, reversible clasping; 2, delayed but irreversible; 3, immediate and irreversible. Tremor: 0, no tremor; 1, slight and intermittent tremor; 2, permanent tremor; 3, moderate or severe tremor. Coat condition: 0, shiny and tidy coat; 1, partly oily or slightly messy coat; 2, oily and slightly messy coat; 3, oily and messy coat. Ledge test: 0, smooth edge displacement; 1, displacement with static periods; 2, displacement with difficulty and hindlimb slipped; 3, forelimb and hindlimb slipped from the edge. The scores obtained in each of the evaluations were summed to obtain a total score representing the general state of the mice and the RTT-phenotype progression.

At seven weeks of age, mice were daily evaluated for three consecutive days in a battery of behavioral tests, including plus maze, open field, hanging wire, and elevated dowel test to determine the RTT-like behavior progression, as previously described [43,46] and briefly described as follows:

Plus Maze: To evaluate anxiety-like behavior and spatial perception, mice were placed in the center of an elevated maze with a cross of two open and two closed arms (homemade). The time spent in the maze’s arms or center was recorded. Open Field: To evaluate anxiety-like behavior, motor rearing, and exploratory activity, mice were placed in an open arena with a photo beam system (Med Associates Inc., St. Albans, VT, USA). Mice were placed at the center of the arena, and their activity was recorded during 30 min at 10 min intervals. Hanging wire: To evaluate forepaw strength, the hanging wire test was performed by hanging mice by their forepaws from a suspended wire, and the number of falls in two minutes was recorded. Elevated dowel: To evaluate motor coordination, mice were assessed in a dowel apparatus consisting of two 70 cm elevated platforms enclosed by 7 cm high walls except for one side that connects both elevated platforms by a 70 cm long dowel of 0.7 cm radius. As described previously [43], mice were habituated by 1 min to each platform, then received a short training placing them on the dowel 10 cm away from one of the platforms. After habituation, mice were placed in the middle of the dowel, and the latency to start moving, time of the first arrival, the number of arrivals in a period of 90 s, and the total number of falls were recorded.

At eight weeks of age, mice were subjected to a rotarod test for 4 consecutive days. Rotarod: To evaluate motor coordination and motor learning, mice were evaluated on an accelerating rotarod for 4 consecutive days, with 4 trials per day and 30-min rest intervals. The rotarod was configurated in accelerating mode, starting at 5 rpm and increasing to 20 rpm over 180 s, and maintained for a maximum of 300 s of total training time. Mice were placed on the rotating cylinder, and the time to fall was recorded. 

### 2.3. Golgi Staining and Morphological Evaluation

To determine the effect of environmental enrichment on motor cortical cytoarchitecture, 3-week-old or 7-week-old mice were deeply anesthetized and then transcardiac perfused with cold saline, followed by 4% PFA. After that, the brains were removed from the skull and immersed in Golgi impregnation solution according to the manufacturer’s instructions for the FD Rapid GolgiStain^TM^Kit. The brains were sliced with a vibratome at 200 µm before final staining, and the stained sections were mounted on slides with Vectamount. Pyramidal neurons of layer V of motor cortex M1-M2 were drawn under a microscope, emulating the neurolucida system. Neuronal soma were included in the analysis if they had a characteristic shape, were in the plane of the slide, and had the dendritic tree in the thick of the slide. Sholl analysis was then performed using an Image J plugin with concentric circles of 20 µm. To determine the dendritic spine density, 2nd and 3rd-order dendrites were selected, and pictures were taken using an Olympus IX-71 microscope (Olympus, Hamburg, Germany) connected to an MSHOT digital camera. Images were analyzed with the software, and dendritic density was evaluated using the ImageJ2 plugin version: 2.14.0/1.54f, Analyze Skeleton, in a dendritic fragment of 30 µm. 

### 2.4. RNA-Seq and Gene Ontology Enrichment Analysis

Brains from 7-week-old WT and *Mecp2^-/y^* mice were dissected. According to the manufacturer’s instructions, RNA was isolated from the forebrain using TRIzol (Invitrogen, Waltham, MA, USA). A pool of four RNA samples per condition (approximately 4 μg of WT RNA and 4 ug of *Mecp2^-/y^* RNA) was sent to Macrogen Co., Ltd. (Seoul, Republic of Korea) as a single replicate for quality control of total RNA integrity using an Agilent Technologies 2100 Bioanalyzer (Agilent RNA 6000 nano kit (Agilent, Santa Clara, CA, USA, cat.# 5067-1511)) and microarray analysis. Basic statistics (fold change, group mean, standard deviation), identification of differentially expressed genes (T-test, LPE test, ANOVA with *p*-value < 0.05), and multiple testing correction (Fold Discovery Rate, Bonferroni with adjusted *p*-value < 0.05) were carried out by the Analysis Service of Macrogen Co., Ltd.

Gene Ontology Enrichment Analysis was performed for differentially expressed genes using the ClueGO app in Cytoscape, _v3.8.2 with the following reference database: GO_ImmuneSystemProcess-EBI-UniProt-GOA-ACAP-ARAP_25.05.2022_00h00: 3113. Default parameters were used.

### 2.5. Gene and Protein Expression

Gene expression: Brains were dissected from 7-week-old mice exposed to environmental enrichment or control cages for 5 weeks. RNA was isolated from the motor cortex and reverse transcribed as previously described [51]. Briefly, the motor cortex was dissected, and samples were homogenized in TRIzol (Invitrogen) according to the manufacturer’s instructions. RNA was precipitated and treated with one unit of DNase I (Life Technologies, Carlsbad, CA, USA). Five micrograms of total RNA were reverse transcribed using random primers and the ImProm II kit (Promega, Madison, WI, USA). cDNA was quantified by qPCR using Kapa SYBR Quantimix (Kapa). The qPCR analysis was performed in triplicates from one reverse transcribed product using the Rotor-Gene 6000 (Corbett). Values were analyzed following the 2^−ΔΔCt^ method using cyclophilin-A (*Cyc*) as a normalizer [52]. The list of primers that were used is described in Table 1. Protein expression: Brains were dissected, and the motor cortex was homogenized using a Douncer tissue grinder in RIPA buffer (Thermo Scientific, Waltham, MA, USA) supplemented with 1x protease inhibitor cocktail (Sigma, Kanagawa, Japan, P8340) and 1x phosphatase inhibitor cocktail (Pierce). Twenty-five micrograms of protein were electrophoresed on 4% and 8–12% SDS polyacrylamide gels, transferred onto nitrocellulose membranes (Bio-Rad, Hercules, CA, USA), and blocked for 1 h at room temperature with freshly prepared TBS-T buffer containing 5% non-fat dry milk. Membranes were incubated overnight with anti-EEAT1 (GLAST) antibody (ab416, Abcam), anti-EEAT2 (GLT-1) antibody (sc-15317, Santa Cruz Biotechnology, Dallas, TX, USA), or β-Actin antibody (sc-47778, Santa Cruz Biotechnology) at 4 °C, washed, and incubated with secondary HRP-conjugated IgG for 2 h at room temperature. Bands were visualized with WESTAR SUPERNOVA Cod. XLS3 (Cyanagen, Bologna, Italy) chemiluminescent substrate according to the manufacturer’s instructions in a Syngene G:Box (Hertfordshire, UK). Densitometry of immunoreactive bands was quantitated using ImageJ software, with the expression of β-Actin as a normalizer.

### 2.6. Collection of Mouse Young Plasma Samples and Plasma Injection

Six-week-old wild-type mice in the C57BL/6 and 129/SvJ genetic backgrounds were deeply anesthetized by an intraperitoneal (IP) injection of Avertin (200 mg/Kg). Blood was extracted by cardiac puncture using a 22 G syringe, and the blood was transferred into a 1.6 mL heparinized tube. The blood samples were then centrifuged for 20 min at 2000× *g*, and the supernatant was centrifuged for 2 min at 14,000× *g* to remove platelets. After that, samples were frozen at −80 °C in 100 µL aliquots containing 50% C57BL/6 and 50% 129/SvJ mouse plasma. A 100 µL aliquot of young plasma or saline solution was IP injected into *Mecp2^-/y^* mice every other day from 4 weeks of age, for a total of 8 injections until 6 weeks of age. 

### 2.7. Diaphragm Neuromuscular Junction

The diaphragm muscle was dissected, and the whole-mount was fixed in 0.5% formaldehyde (FA) in 1X Phosphate Buffered Saline (PBS) at 22 °C for 90 min. Samples were incubated with 0.1 M glycine in 1X PBS, permeabilized with PBST (1X PBS/0.5% TritonX-100), and blocked with 4% goat serum (GS) dissolved in PBST 1 h at 22 °C. Muscles were incubated with 4% GS-PBST containing Alexa488-conjugated α-bungarotoxin (BTX) (Invitrogen, Carlsbad, CA, USA) (1:500) for 12–16 h at 4 °C. The samples were post-fixed with 0.5% FA in 1X PBS for 10 min at 22 °C and subsequently flat-mounted between two coverslips. For endplate band quantification, fluorescent images of α-BTX-stained right hemidiaphragm were captured with a ZEISS Axio Zoom.V16 scope, and AChR clusters distribution were analyzed. The endplate bandwidth of 20–40 bins per animal was measured using ImageJ. 

### 2.8. Statistical Analysis

The software GraphPad PRISM Version 10.2.0 (San Diego, CA, USA) was used for statistical analysis. Data are presented as mean ± SEM values, and differences were analyzed using one-way ANOVA, two-way ANOVA, or Simple survival analysis (Mantel–Cox), as indicated in each figure. Statistical significance was defined as * *p* < 0.05, ** *p* < 0.01, *** *p* < 0.001, **** *p* < 0.0001, and ns for non-statistical differences. 

## 3. Results

### 3.1. The Exposure to a Neuronal Plasticity-Dependent Paradigm Ameliorates the Phenotype Exhibited by a Mouse Model of Rett Syndrome

Rett syndrome is characterized by a decreased neural plasticity, and exposure to experience-dependent paradigms has been shown to increase dendrite complexity in several mouse models of neurological disorders, including RTT [43,50]. To gain insight into the cellular and molecular mechanisms underlying this therapeutic effect, we permanently housed *Mecp2^-/y^* mice and their WT littermate in EE or SC from weaning, when the RTT phenotype, if it is apparent, is mild. We recorded mouse weight and survival weekly, and at 7 weeks of age, the general mouse condition was estimated by using a battery of phenotypic evaluations, including hindlimb clasping reflex and corporal tremor as indicators of neurological deterioration progression, piloerection as an indicator of grooming and social interaction, the ledge test as a measurement of motor coordination, and the total score obtained from the sum of these phenotypic evaluations was used as an indicator of mouse general condition. 

**Figure 1:**
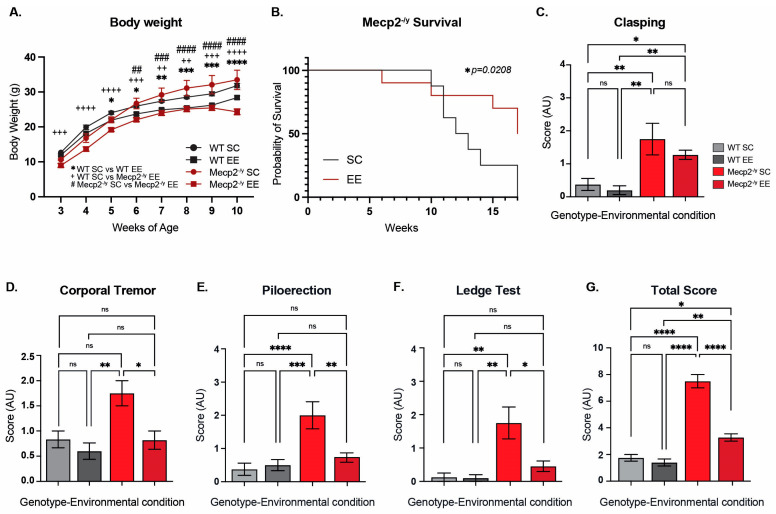
Permanent exposure to an enriched environment (EE) since weaning attenuates the RTT-like phenotype in mice. (**A**) Body weight recordings of WT mice exposed to an enriched environment (EE) or standard cages (SC), and *Mecp2^-/y^* mice exposed to SC or EE. *Mecp2^-/y^* mice exposed to EE showed a decrease in body weight in comparison with *Mecp2^-/y^* mice housed in control conditions. (**B**) Lifespan of *Mecp2^-/y^* mice exposed to SC or EE. The decreased lifespan of *Mecp2^-/y^* mice is extended by exposure to EE. (**C**–**G**) Overall phenotypic evaluation of *Mecp2^-/y^* mice: (**C**) the increased level of clasping shown by *Mecp2^-/y^* mice was not prevented by exposing mice to EE. The increased levels of corporal tremor (**D**), piloerection (**E**), and hind-limb discoordination (**F**) shown by *Mecp2^-/y^* mice were prevented by exposure to EE. (**G**) General state evaluation of mice by a total score, which was higher in *Mecp2^-/y^* mice in SC compared to WT, and diminished by exposure to EE in Mecp2^-/y^ mice. WT *n* = 33; *Mecp2^-/y^ n* = 10–16 biologically independent animals. Data are presented as mean ± SEM values, and differences were analyzed by (**B**) Simple survival analysis Mantel–Cox test, followed by Wilcoxon test, and (**A**,**C**–**G**) two-way ANOVA, followed by Tukey’s multiple comparisons tests. The levels of significance are shown as * *p* < 0.05; ** *p*, ^++^ *p*, or ^##^ *p* < 0.01; ^+++^ *p*, ^###^ *p*, or *** *p* < 0.001; **** *p*, ^++++^ *p*, or ^####^ *p* < 0.0001; ns, non-statistical differences.

Before puberty, *Mecp2^-/y^* mice showed decreased body weight, a phenotype that is inverted after sexual maturity, as demonstrated by our results and consistent with previous literature [5,16,17,53]. *Mecp2^-/y^* mice exposed to EE showed a reduced body weight compared to *Mecp2^-/y^* mice housed in control conditions (Figure 1A), indicating that exposure to EE improves energy homeostasis. Furthermore, the decreased lifespan of *Mecp2^-/y^* mice was shown to be increased by exposure to EE. *Mecp2^-/y^* mice showed a half-life of 12 weeks of age, and no mice survived beyond 16 weeks; however, *Mecp2^-/y^* mice exposed to EE showed a half-life of 17 weeks (Figure 1B). 

To evaluate the effect of EE exposure on the classic RTT phenotype after sexual maturity, mice were assessed using a battery of general condition tests. As expected, *Mecp2^-/y^* mice showed an increased level of clasping compared to WT mice, but this neurological phenotype was not prevented by exposing mice to EE (Figure 1C). Despite this result, exposure to EE prevented the other phenotypic parameters evaluated. *Mecp2^-/y^* mice showed increased levels of corporal tremor, piloerection, and hindlimb discoordination compared to WT mice; however, *Mecp2^-/y^* mice exposed to EE showed a reduction in these phenotypes, which was not different from WT mice, in contrast to *Mecp2^-/y^* mice in SC (Figure 1D–F). As an evaluation of the general mice condition, the score of each test was summed to obtain a total phenotypic evaluation score, which was, as expected, higher in *Mecp2^-/y^* mice in comparison to WT mice; however, this parameter was rescued in *Mecp2^-/y^* mice exposed to EE compared to *Mecp2^-/y^* mice in SC. Together, these results show that permanent exposure to an EE since weaning improves energy homeostasis, increases lifespan, and attenuates the RTT-like phenotype. 

### 3.2. The Neuronal Plasticity-Dependent Paradigm Exposure Reduces the Behavioral Alterations and Motor Deficits Exhibited by a Mouse Model of Rett Syndrome

**Figure 2:**
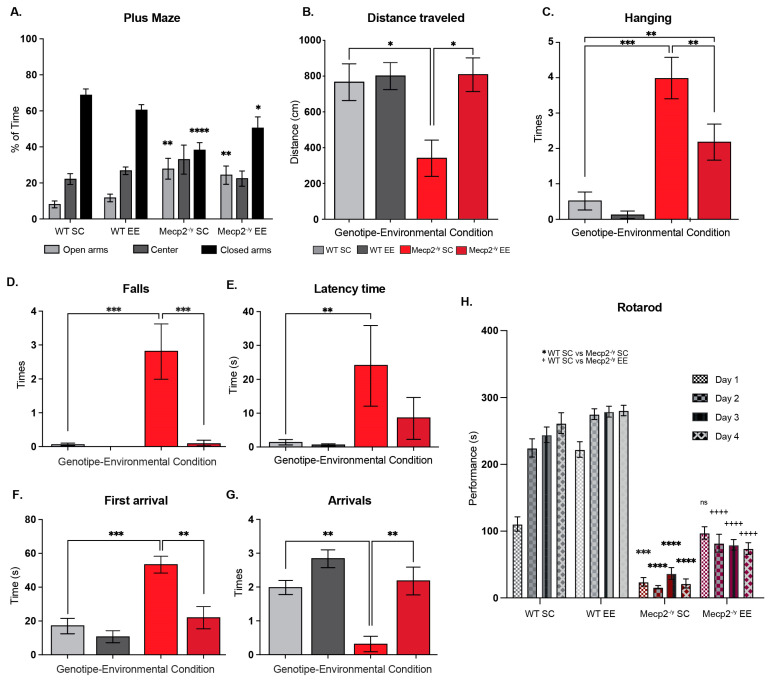
Exposure to a neuronal plasticity-dependent paradigm to increase neuronal plasticity attenuates the behavioral phenotype and motor deficits in an RTT mouse model. (**A**) Elevated plus maze to evaluate anxiety-like behavior; the exposure to EE reestablished the preference for closed arms in *Mecp2^-/y^* mice. (**B**) Traveled distance in open field test, as a locomotion parameter; *Mecp2^-/y^* mice exposed to EE showed improved locomotion activity compared to *Mecp2^-/y^* mice exposed to SC. (**C**) Wire-hanging test to evaluate motor function; *Mecp2^-/y^* mice exposed to EE showed less failure in hanging from the wire in comparison with *Mecp2^-/y^* mice in SC. (**D**–**G**) Elevated dowel test to evaluate the motor function, coordination, and control; (**D**) *Mecp2^-/y^* mice in EE showed a similar number of falls as WT mice and fewer falls than *Mecp2^-/y^* mice in SC; EE exposure improved latency time (**E**) and the time of the first arrival (**F**) in *Mecp2^-/y^* mice since they were similar to those exhibited by WT mice. (**G**) The number of arrivals of *Mecp2^-/y^* mice in SC was less than in WT mice, and this phenotype was completely prevented by exposure to EE. (**H**) Motor coordination and learning were evaluated on the rotating cylinder of a rotarod; the poor motor coordination in *Mecp2^-/y^* mice was prevented by exposure to EE; however, motor learning was not recovered. WT *n* = 13–21; *Mecp2^-/y^ n* = 6–10 biologically independent animals. Data are presented as mean ± SEM values, and differences were analyzed by two-way ANOVA followed by Tukey’s multiple comparisons tests. The levels of significance are shown as * *p* < 0.05, ** *p* < 0.01, *** *p* < 0.001, **** *p* < 0.0001, and ^++++^ *p* < 0.0001.

The next step was to evaluate whether exposure to EE could attenuate the behavioral phenotype of the RTT mouse model. *Mecp2^-/y^* mice and their WT littermates were evaluated using a second battery of phenotypic evaluations to assess anxiety-like behavior, spatial perception, locomotion, and motor coordination performance. First, mice were evaluated in the elevated plus maze. As expected, WT mice showed a marked preference for staying in the closed arms of the maze. As previously demonstrated [43], *Mecp2^-/y^* mice exposed to SC showed a higher preference for open arms and, on the contrary, less preference for closed arms, with no preference for any of the arms. EE exposure did not affect the arm preference in WT mice; however, in *Mecp2^-/y^* mice, EE reestablished the preference for closed arms (Figure 2A). Mice were then evaluated in the open field test. We measured traveled distance as a locomotion parameter. *Mecp2^-/y^* mice exposed to SC showed significantly less locomotion activity than WT mice exposed to SC and EE. However, *Mecp2^-/y^* mice exposed to EE showed increased locomotor activity compared to *Mecp2^-/y^* mice exposed to SC (Figure 2B). Thus, EE exposure improved locomotion in an RTT mouse model.

A distinctive symptom of RTT patients is reduced muscle tone and motor control. Therefore, mice were evaluated in the wire-hanging test to assess whether EE exposure improves motor strength. As expected, *Mecp2^-/y^* mice fail more often when hanging from an elevated wire than WT mice in SC. *Mecp2^-/y^* mice exposed to EE showed less failure when hanging from the wire than *Mecp2^-/y^* mice in SC, though higher than that observed in WT mice (Figure 2C). To determine the effect of EE exposure on motor coordination, function, and control, the performance of WT and RTT mice was evaluated in the elevated dowel test for 60 s. *Mecp2^-/y^* mice in SC showed a higher number of falls in comparison to WT mice, and this phenotype was entirely prevented by EE exposure since *Mecp2^-/y^* mice in EE showed a similar number of falls than WT mice and fewer falls than *Mecp2^-/y^* mice housed in SC (Figure 2D). The latency of mice to start moving and the time of first arrival were also recorded. The results show that *Mecp2^-/y^* mice in SC started moving later than WT mice and took longer to reach the safe elevated platform. EE exposure improved these parameters in *Mecp2^-/y^* mice since the latency time and the time of the first arrival were similar to those exhibited by WT mice, and in the case of the first arrival, it was less than in *Mecp2^-/y^* mice in SC (Figure 2E,F). Following these results, the number of arrivals of *Mecp2^-/y^* mice in SC was less than in WT mice, and this phenotype was prevented entirely by EE exposure since *Mecp2^-/y^* mice in EE showed a similar number of arrivals as the WT mice and higher than *Mecp2^-/^*^y^ mice in SC (Figure 2G). Motor coordination was also evaluated on the rotating cylinder of a rotarod, which is also used to assess motor learning. *Mecp2^-/y^* mice showed poor motor coordination and failure to stay on the rotating cylinder compared to WT mice. This motor phenotype was prevented entirely by exposure to EE since *Mecp2^-/y^* mice exposed to EE showed a motor phenotype comparable to WT mice in SC on the first training day. Despite this motor improvement, EE exposure failed to induce motor learning in *Mecp2^-/y^* mice (Figure 2H). 

### 3.3. The Exposure to a Neuronal Plasticity-Dependent Paradigm Decreases the Synaptic Deficit Exhibited by an RTT Mouse Model

**Figure 3:**
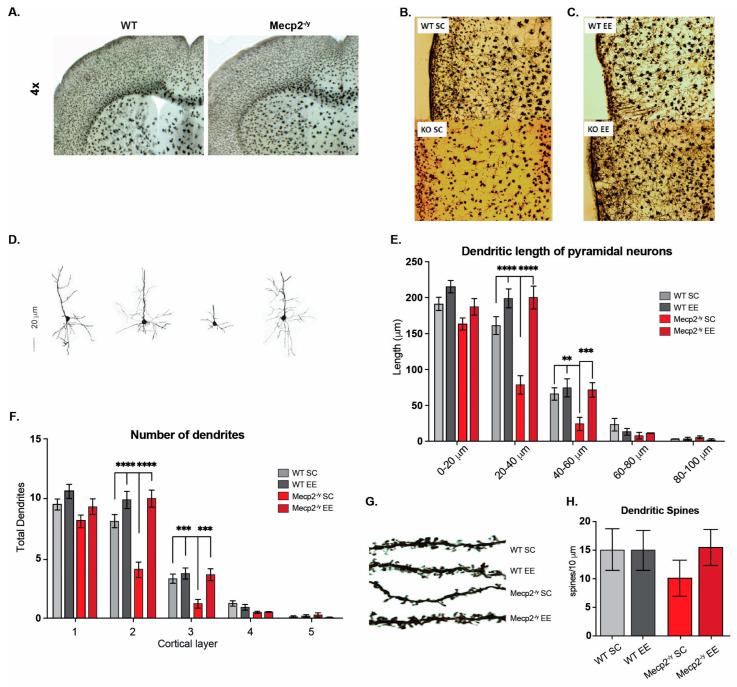
Exposure to a neuronal plasticity-dependent paradigm decreases the synaptic deficit exhibited by an RTT mouse model. (**A**–**D**) M1-M2 motor cortex cytoarchitecture at 3 and 7 weeks of age in WT and *Mecp2^-/y^* mice exposed to either control or EE housing. (**A**) At 3 weeks of age, the motor cortex of *Mecp2^-/y^* mice is not significantly different from WT. (**B**,**C**) Reduction of neuronal complexity and moderate astrogliosis in *Mecp2^-/y^* mice at 7 weeks of age, which was improved by exposure to EE. (**D**,**E**) Evaluation of the dendritic length of pyramidal neurons of cortical layer 5, which was reduced *in Mecp2^-/y^* mice in SC in comparison with WT mice, and this reduction was prevented by EE exposure. (**F**) The number of dendrites in pyramidal neurons was evaluated. *Mecp2^-/y^* mice exposed to EE had a similar number to that of WT mice. (**G**,**H**) The dendritic spine density of second-order dendrites of pyramidal neurons from the motor cortex was evaluated. *Mecp2^-/y^* mice exhibit less spine density in comparison to that of WT in SC, which was prevented by EE exposure. WT *n* = 30–50; *Mecp2^-/y^ n* = 40 neurons. Data are presented as mean ± SEM values, and differences were analyzed by two-way ANOVA followed by Tukey’s multiple comparisons tests. The levels of significance are shown as ** *p* < 0.01, *** *p* < 0.001, and **** *p* < 0.001.

Since the general condition of the mice and behavioral tests showed that EE exposure improved motor performance in *Mecp2^-/y^* mice, we next evaluated the motor cortex phenotype. To that end, we compared the M1-M2 motor cortex cytoarchitecture at 3 and 7 weeks of age in WT and *Mecp2^-/y^* mice exposed to SC or EE housing. We chose these ages as critical times in RTT phenotype progression. While the RTT phenotype is barely apparent in mice at 3 weeks of age, at 7 weeks of age, motor performance is diminished in *Mecp2^-/y^* mice in SC, and in response to EE, an improvement was observed at this age. At 3 weeks of age, the motor cortex of *Mecp2^-/y^* mice is not significantly different from WT mice (Figure 3A). However, at 7 weeks of age, the *Mecp2^-/y^* motor cortex seems disorganized, with reduced neuronal complexity and signs suggesting astrogliosis (Figure 3B). Nevertheless, the motor cortex of *Mecp2^-/y^* mice exposed to EE seems similar to WT mice (Figure 3C), with an apparent better organization, improved neuronal complexity, and no signs of astrogliosis, which contrasts with that observed in *Mecp2^-/y^* mice in SC (Figure 3B,C). 

The dendritic length of cortical layer V pyramidal neurons was evaluated to compare this observation. The results show a reduction in the dendritic length of pyramidal neurons from *Mecp2^-/y^* mice in SC compared to those of WT mice. However, this difference was not observed in the dendritic length of *Mecp2^-/y^* mice exposed to EE (Figure 3D,E). The number of dendrites was also evaluated, and similar results were found. The pyramidal neurons of the motor cortex from *Mecp2^-/y^* mice in SC exhibit decreased dendrites compared to those from WT mice. Nevertheless, the number of dendrites in pyramidal neurons of *Mecp2^-/y^* mice exposed to EE was similar to that of WT mice in SC (Figure 3F). These morphological changes related to neuronal plasticity were similar when the dendritic spine density was evaluated. Second-order dendrites of pyramidal neurons from the motor cortex of *Mecp2^-/y^* mice exhibit less spine density than those from WT mice in SC. However, in *Mecp2^-/y^* mice exposed to EE, the dendritic spine density was similar to that of WT mice in SC and higher than that of *Mecp2^-/y^* mice in SC (Figure 3G,H). These results show that early EE exposure increases structural neuronal plasticity markers and prevents the cortical impairment associated with RTT neurological progression. 

### 3.4. The Expression of Genes Related to Cellular Homeostasis Is Altered in an RTT Mouse Model and Can Be Partially Reestablished by Exposure to a Neuronal Plasticity-Dependent Paradigm

**Figure 4:**
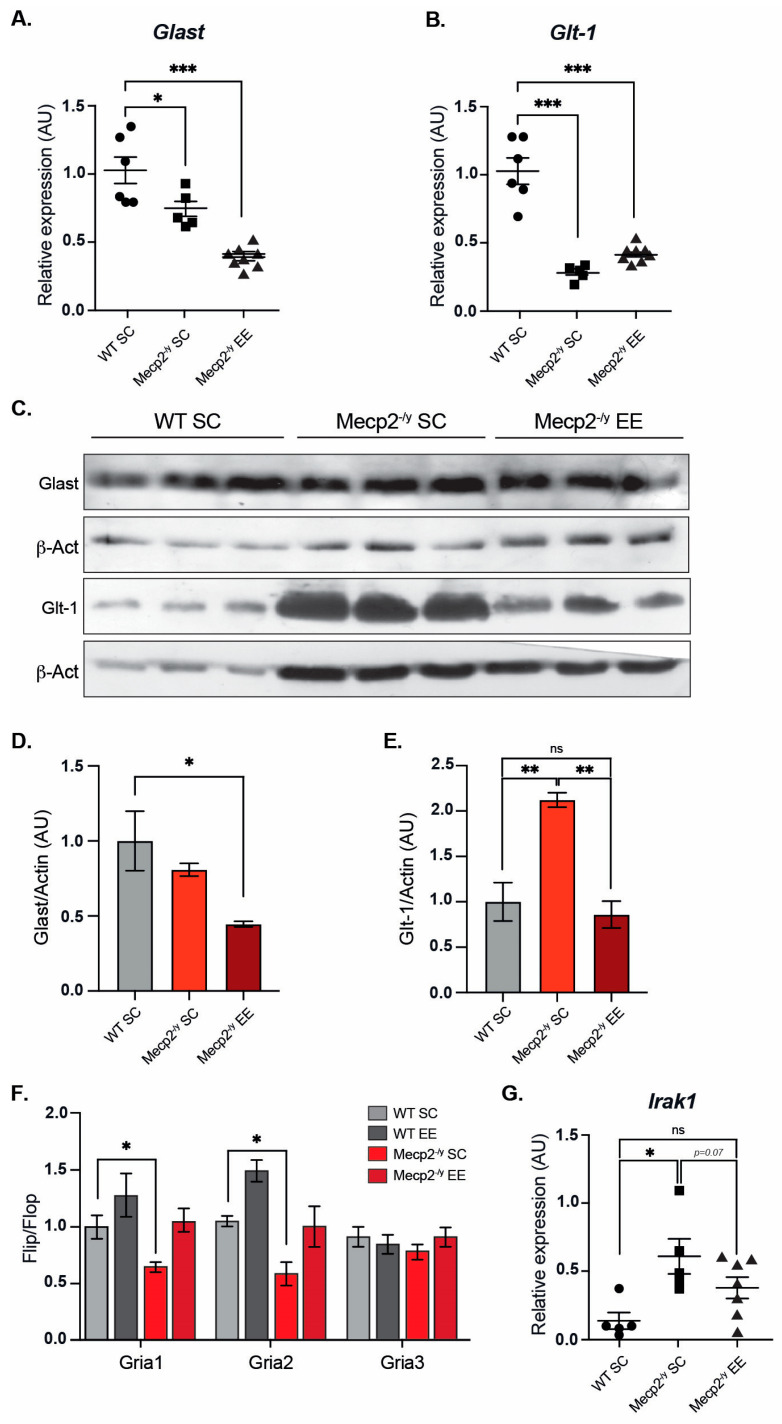
Exposure to a neuronal plasticity-dependent paradigm can partially reestablish expression levels of genes related to cellular homeostasis. mRNA expression of *Glast* (**A**) and *Glt-1* (**B**) by RT-qPCR in WT mice exposed to SC, and *Mecp2^-/y^* mice exposed to SC or EE. EE exposure failed to reestablish *Glast* and *Glt-1* expression levels in *Mecp2^-/y^* mice. (**C**,**D**) Glast protein levels are similar in *Mecp2^-/y^* mice housed in SC and WT mice and decreased in *Mecp2^-/y^* mice exposed to EE. (**C**–**E**) Glt-1 protein levels are increased in *Mecp2^-/y^* mice housed in SC compared to WT mice; EE exposure reestablishes Glt-1 protein levels in *Mecp2^-/y^* mice. (**F**) RT-qPCR of flip and flop splicing variants of *Gria1*, *Gria2,* and *Gria3* genes shows that EE exposure restores the flip/flop ratio of *Gria1* and *Gria2* in *Mecp2^-/y^* mice. (**G**) mRNA expression of *Irak1* by RT-qPCR; EE exposure reduced *Irak1* expression in *Mecp2^-/y^* mice to levels similar to WT mice. WT *n* = 3–8; *Mecp2^-/y^ n* = 3–8 biologically independent animals. Data are presented as mean ± SEM values, and differences were analyzed by (**A**,**B**,**D**,**E**,**G**) one-way ANOVA followed by Tukey’s multiple comparisons tests or (**F**) two-way ANOVA followed by Tukey’s multiple comparisons tests. The levels of significance are shown as * *p* < 0.05, ** *p* < 0.01 and *** *p* < 0.001; ns, non-statistical differences.

It has been demonstrated that reactivation of Mecp2 neuronal expression can rescue the RTT phenotype in mice [54], supporting the idea that Mecp2 deficiency in neurons is sufficient to cause an RTT phenotype. However, in vitro evidence indicates that Mecp2 deficiency in glia may also contribute to brain dysfunction. Mecp2-deficient microglia cause dendritic and synaptic damage mediated by elevated glutamate (Glu) release [55]. Mecp2 is involved in Glu clearance through the regulation of Glu transporters (Glast/Eaat1 and Glt-1/Eaat2) and Glutamine Synthetase in astrocytes. Glu clearance and production are abnormal in *Mecp2*-deficient astrocytes, probably contributing to the pathological process of RTT [56]. Since mRNA expression of *Glast* and *Glt-1* glutamate transporters is decreased in *Mecp2*-null astrocytes [56], we evaluated whether EE exposure can reestablish either *Glast* or *Glt-1* mRNA expression in the motor cortex of *Mecp2^-/y^* mice. The results show that EE exposure failed to restore mRNA levels of *Glast* (Figure 4A) and *Glt-1* (Figure 4B) to WT levels. When we evaluated protein levels of Glast and Glt-1 in WT and *Mecp2^-/y^* mice exposed to SC and *Mecp2^-/y^* mice exposed to EE (Figure 4C–E), we found that Glast protein levels behave as its mRNA expression, they decreased in *Mecp2^-/y^* mice in SC housing, and EE exposure did not restore Glast protein levels (Figure 4C,D). However, contrary to what was expected, Glt-1 protein levels are increased in *Mecp2^-/y^* mice exposed to SC, and EE exposure decreased Glt-1 protein levels in *Mecp2^-/y^* mice to WT levels (Figure 4C,E). These results indicate that in *Mecp2^-/y^* mice, exposure to EE decreases the elevated levels of Glt-1 by a mechanism not associated with transcriptional control of its coding genes. This is very relevant for glutamate availability in the motor cortex, where Glt-1 is the most abundantly expressed glutamate transporter [57].

The proper control of the motor cortex involves glutamatergic neurotransmission. Alternative splicing in the extracellular ligand binding domain of AMPA receptors (AMPAR) generates two variants— flip and flop. The flop variant of the *Gria2* gene, coding for the GluA2 subunit of the AMPA receptor, promotes the channel to close more rapidly, thus desensitizing the channel faster than the flip sequence [58]. It has been previously reported that the loss of Mecp2 affects the flip/flop splicing of AMPAR genes, leading to a significant splicing shift in the flip/flop exon toward the flop inclusion [59]. We evaluated the flip/flop ratio of *Gria1*, *Gria2*, and *Gria3* genes that encode GluA1, GluA2, and GluA3 AMPAR subunits, respectively. As expected, we found a significant decrease in *Gria1* and *Gria2* flip/flop ratios in *Mecp2^-/y^* mice exposed to SC, which was counteracted by exposure to EE (Figure 4F). 

To gain insight into molecular mechanisms altered in the forebrain of *Mecp2^-/y^* mice, a single-replicate microarray analysis of forebrain samples from *Mecp2^-/y^* mice versus WT mice was performed to seek potential candidate genes whose expression could be re-established by EE exposure. The complete list of differentially expressed genes is shown in Supplementary File S1. We performed an immune-related gene ontology (GO) enrichment analysis for all differentially expressed genes in *Mecp2^-/y^* mice compared with WT mice. We found the following enriched terms, mainly upregulated in *Mecp2^-/y^* mice: “Toll-like receptor 4 signaling pathway” (GO:0034142), “Response to type I interferon” (GO:0034340), and “Cellular response to type I interferon” (GO:0071357), with *Irak1* belonging to these inflammatory GO terms (Figure S1). As previously reported, we found that *Irak1* mRNA levels were higher in the forebrain of *Mecp2^-/y^* mice. Increased aberrant expression of *Irak1* has been shown to cause increased NF-κB activity in *Mecp2^-/y^* mice [60], and given its role in inflammation, we further evaluated *Irak1* mRNA expression in WT mice exposed to SC using RT-qPCR, and *Mecp2^-/y^* mice both exposed to SC and EE (Figure 4G). As expected, *Mecp2^-/y^* mice exposed to SC showed increased *Irak1* expression. However, *Irak1* expression in *Mecp2^-/y^* mice exposed to EE did not differ from WT mice exposed to SC. These results show that exposure to EE attenuates the molecular phenotype of *Mecp2^-/y^* mice associated with neurotransmission in the forebrain, such as Glu transporter levels, *Gria* splicing, and inflammatory process, potentially contributing to the attenuated progression of the motor phenotype.

### 3.5. The Exposure to a Neuronal Plasticity-Dependent Paradigm Ameliorates the Altered Distribution of Neuromuscular Synapses in an RTT Mouse Model

Respiratory impairment, including hyperventilation, periods of breath-holding, forced breathing, and apneas, is one of the leading causes of premature death in RTT patients [7,9]. An attractive possibility to explain the increased lifespan and enhanced motor capacity observed in response to EE is improved neuromuscular communication, leading to enhanced skeletal muscle activity. Thus, we next evaluated the distribution of neuromuscular synapses. To this end, we used the diaphragm, a key respiratory muscle that contracts in response to phrenic nerve activity to allow breathing.

**Figure 5:**
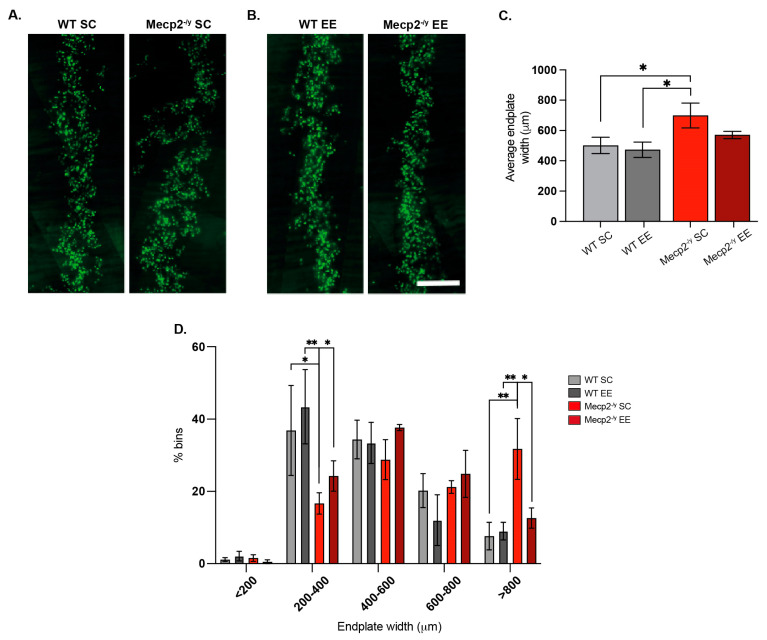
Exposure to a neuronal plasticity-dependent paradigm ameliorates the alterations in neuromuscular junction distribution exhibited by an RTT mouse model. (**A**,**B**) Representative images of endplate distribution in the respiratory diaphragm muscle from WT and *Mecp2^-/y^* mice exposed to SC (**A**) or EE (**B**). (**C**) *Mecp2^-/y^* mice exposed to SC had increased average endplate width phenotype, which was not observed in *Mecp2^-/y^* mice exposed to an enriched environment. (**D**) Histogram distribution of different endplate width ranges shows that the relative abundance of endplates from WT mice was not affected by the housing conditions; however, in *Mecp2^-/y^* mice exposed to EE, the proportion of wider endplates (>800 μm width) was significantly reduced. WT *n* = 3; *Mecp2^-/y^ n* = 3 biologically independent animals. Data are presented as mean ± SEM values, and differences were analyzed by (**C**) one-way ANOVA followed by Kruskal–Wallis’s multiple comparisons tests or (**D**) two-way ANOVA. The levels of significance are shown as * *p* < 0.05; ** *p* < 0.01.

The diaphragm of *Mecp2^-/y^* mice housed in SC exhibited a wider endplate distribution than WT mice (Figure 5A,C), a phenotype associated with defects in neuromuscular junction assembly [61]. Remarkably, while EE exposure did not affect neuromuscular junction distribution in the diaphragm of WT mice, in *Mecp2^-/y^* mice, EE exposure rescued the phenotype (Figure 5B,C). Indeed, quantification shows that the relative abundance of wider endplates (>800 μm width) was significantly reduced after EE exposure in *Mecp2^-/y^* mice, reaching values comparable to those in WT mice (Figure 5D). These findings demonstrate that a neuronal plasticity-dependent paradigm can induce changes in the distribution of the peripheral neuromuscular synapse. These changes likely contribute to the improved neurological and motor phenotypes and the increased lifespan observed in RTT mice in response to EE. 

### 3.6. The Treatment with Plasma from Young Mice Attenuates the RTT-like Phenotype in Mice

Our results showed that exposure to a neuronal plasticity-dependent paradigm induces neurological and motor improvement in an RTT mouse model associated with central and peripheral structural changes in neurons and at the neuromuscular junction. One of the components of our experimental paradigm is motor activity, which is reduced in *Mecp2^-/y^* mice. Additionally, it has been demonstrated that physical activity increases plasma levels of neurotrophic factors [62], and that treatment with plasma from young increases neuronal plasticity in aged mice [49]. We hypothesize that the increased physical activity induced by EE in *Mecp2^-/y^* mice could modify plasma components with neurological effects. Therefore, treatment with young plasma from WT mice with a similar physical activity level to *Mecp2^-/y^* mice could, at least partially, emulate the beneficial effect of exposure to an EE. 

**Figure 6:**
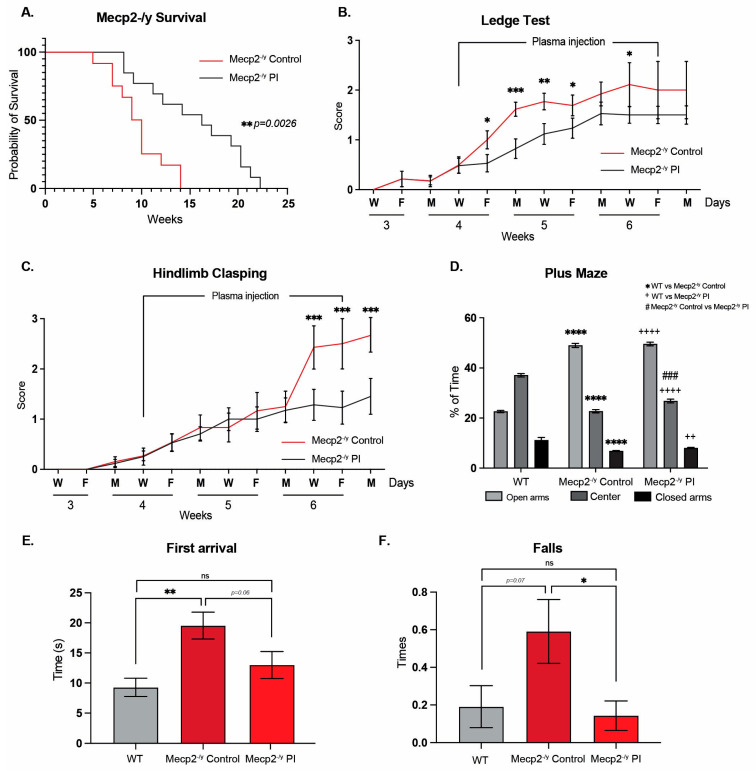
Treatment with plasma from young mice attenuates the RTT phenotype in a mouse model of the disease. (**A**) Lifespan analysis of *Mecp2^-/y^* mice with or without IP plasma injection; IP plasma injection increased the survival of *Mecp2^-/y^* mice. (**B**,**C**) Phenotypic evaluation of *Mecp2^-/y^* mice; IP plasma injection attenuated the progression of motor and neurological RTT-like symptoms, as evaluated by the (**B**) ledge test and (**C**) hindlimb clasping. (**D**) In the elevated plus maze, used to evaluate anxiety-like behavior, IP plasma treatment did not prevent the increased preference of *Mecp2^-/y^* mice for the open arm of the maze. (**E**,**F**) In the elevated dowel test, used to evaluate motor coordination, IP plasma treatment prevented the increased time to first arrival (**E**) and the increased number of falls (**F**) in *Mecp2^-/y^* mice. WT *n* = 22; *Mecp2^-/y^ n* = 6–10 biologically independent animals. Data are presented as mean ± SEM values, and differences were analyzed by (**A**) Simple survival analysis Mantel–Cox test followed by the Wilcoxon test and (**B**–**F**) two-way ANOVA followed by Tukey’s multiple comparisons tests. The levels of significance are shown as * *p* < 0.05; ** *p* or ^++^ *p* < 0.01; *** *p* or ^###^ *p* < 0.001; and **** *p* or ^++++^ *p* < 0.001; ns, non-statistical differences.

To test whether treatment with young plasma may decrease the neurological progression and behavioral phenotype exhibited by an RTT mouse model, IP plasma injections started at 4 weeks of age, when mice exhibit a stronger phenotype compared to that at 3 weeks of age. As with EE, IP plasma injection increases *Mecp2^-/y^* mice lifespan (Figure 6A) and attenuates the progression of motor and neurological RTT-like symptoms, as evaluated weekly using the ledge test (Figure 6B) and hindlimb clasping (Figure 6C). To determine whether IP plasma injection recapitulated the elevated plus maze phenotype, we compared the place preference among WT, *Mecp2^-/y^* control mice, and those treated with IP plasma transference. As described above, *Mecp2^-/y^* mice stayed longer in the open arm of the maze in comparison to their WT littermate, a phenotype that was not prevented by IP young mouse plasma transfer treatment (Figure 6D). However, as observed with exposure to EE, the motor coordination phenotype exhibited by *Mecp2^-/y^* mice was prevented by IP injection of young mouse plasma. Similar to that described above, *Mecp2^-/y^* mice showed an increased time of first arrival and an increased number of falls in the dowel test. IP injection of young mouse plasma prevented both phenotypes, reducing the time to first arrival and the number of falls (Figure 6E,F). These results show that the treatment with plasma from young WT mice, which have regular locomotor activity, prevents the motor phenotype of *Mecp2^-/y^* mice.

### 3.7. The Treatment with Plasma from Young Mice Decreases the Synaptic Deficit Exhibited by an RTT Mouse Model

**Figure 7:**
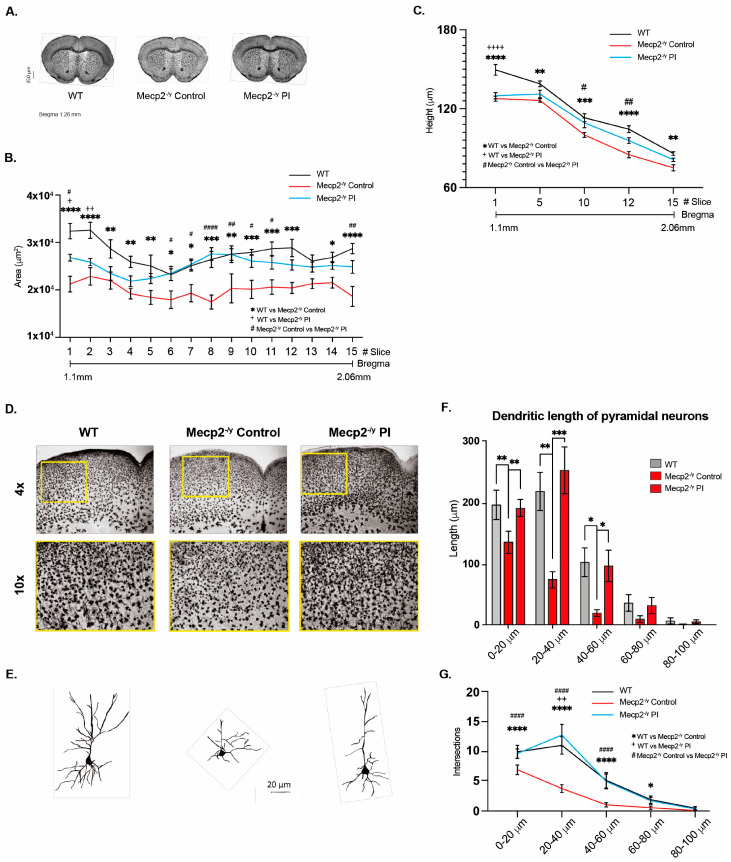
Treatment with plasma from young mice decreases the synaptic deficit exhibited by an RTT mouse model. (**A**) Golgi staining of *Mecp2^-/y^* control and IP plasma injection-treated mice and their WT littermates. Plasma treatment prevented the diminished brain architecture observed in *Mecp2^-/y^* mice in the control group. IP plasma treatment almost reestablished the thickness of the corpus callosum (**B**) and motor cortex (**C**) to that observed in the WT littermates. (**D**) Moderate signs of astrogliosis were observed in *Mecp2^-/y^* mice, which was attenuated by IP plasma injection. (**E**) Neuronal cytoarchitecture of pyramidal neurons was reestablished, as evaluated by dendritic length (**F**) and dendritic arborization complexity (**G**). WT *n* = 5–10; *Mecp2^-/y^ n* = 5–10 biologically independent animals (**B**,**C**). WT *n* = 60; *Mecp2^-/y^ n* = 60 neurons (**F**,**G**). Data are presented as mean ± SEM values, and differences were analyzed by two-way ANOVA followed by Tukey’s multiple comparisons test. The levels of significance are shown as * *p*, ^+^ *p*, or ^#^ *p* < 0.05; ** *p*, ^++^ *p*, or ^##^ *p* < 0.01; *** *p* < 0.001; and **** *p*, ^++++^ *p*, or ^####^ *p* < 0.001.

To determine whether IP injection of young mouse plasma affects brain and neuronal cytoarchitecture, we performed Golgi staining on brain tissue from control mice and IP plasma-treated *Mecp2^-/y^* mice and compared them to their WT littermates (Figure 7A). Systemic therapeutical interventions such as IP injection of young mouse plasma impact the brain and neuronal cytoarchitecture. We observed that the diminished brain architecture observed in *Mecp2^-/y^* mice in the control group was prevented by IP plasma injection, almost reestablishing the thickness of the corpus callosum (Figure 7B) and motor cortex (Figure 7C) like that observed in the WT littermate. In addition, as described above, the motor cortex of *Mecp2^-/y^* mice showed signs of moderate astrogliosis, which was attenuated by IP plasma injection (Figure 7D). Moreover, the neuronal cytoarchitecture of pyramidal neurons of *Mecp2^-/y^* mice was reestablished by IP plasma injections (Figure 7E), as evaluated by dendritic length (Figure 7F) and dendritic arborization complexity (Figure 7G). All these results show that the evaluated neurological parameters reduced in *Mecp2^-/y^* mice are prevented by treatment with plasma from young mice. 

## 4. Discussion

Environmental enrichment (EE) has been widely used to ameliorate impaired neuronal function in various rodent models of brain disorders [38,40,63], presenting a potential alternative or complement to pharmacological treatments of neurodevelopmental disorders. In this study, we show how EE and treatment with young plasma decrease the progression of the Rett Syndrome phenotype in a mouse model of the disease. First, we evaluate the contribution of EE in the phenotype exhibited by *Mecp2^-/y^* mice. Body weight and lifespan were ameliorated by EE exposure since weaning. These results show that exposure to EE might be impacting the energy balance of mice lacking the expression of Mecp2. The role of Mecp2 as a master regulator of body weight has been explored by our group and others [51,53,64,65], and its expression in *Mecp2^-/y^* mice reestablishes increased body weight balance and lifespan [46]. However, the above effect of EE is independent of the expression of Mecp2, indicating that exposure to EE could be activating mechanisms downstream of Mecp2 expression, which could partly compensate for its deficiency.

Further evaluation of RTT phenotype showed that parameters like corporal tremor, piloerection, and hind-limb discoordination, which were increased in *Mecp2^-/y^* mice, were prevented by early EE exposure. The overall phenotypic evaluation of *Mecp2^-/y^* mice, measured as a total score, showed that permanent exposure to EE since weaning attenuates most of the RTT phenotype. However, EE exposure was ineffective in preventing the increased clasping exhibited by *Mecp2^-/y^* mice, indicating that not all neurological functions are targeted by the EE downstream mechanism. On the other hand, behavioral phenotypes like hypoactivity and abnormalities in locomotion, stereotypies, and anxiety reminiscent of the clinical condition have been reported in *Mecp2* mouse mutants [18,53,66,67]. Here, we performed a battery of behavioral tests to determine whether EE exposure improves the clinical-like manifestation already reported. We and others have previously reported that *Mecp2^-/y^* mice exhibited behaviors that correlate with reduced anxiety in the elevated plus maze assay [46,68]. As expected, *Mecp2^-/y^* mice exposed to SC spend more time in the open arms, with no preference for the closed arms as WT mice, and EE exposure reestablished the preference for closed arms in *Mecp2^-/y^* mice. It is still unknown whether this effect of EE is related to the re-establishment of anxiety-associated behavior as a consequence of improving spatial perception. When we evaluated locomotion by measuring the distance traveled in an open field test, *Mecp2^-/y^* mice exposed to EE showed improved locomotion activity compared to *Mecp2^-/y^* mice exposed to SC. Results pointing in the same direction were found when motor control strength and coordination were evaluated, showing that EE exposure prevented the phenotype exhibited by *Mecp2^-/y^* mice. *Mecp2^-/y^* mice exposed to EE had fewer failures when hanging from the wire, fewer falls in the elevated dowel test, and improved latency time and the time of the first arrival compared to *Mecp2^-/y^* mice exposed to SC. Moreover, the number of arrivals was reestablished, exhibiting a performance comparable to that of WT mice. Motor coordination and learning were assessed on the rotating cylinder of a rotarod. Early EE exposure improved poor motor coordination in *Mecp2^-/y^* mice but failed to ameliorate motor learning. All these results showed that early exposure to EE positively impacts counteracting abnormalities in muscle strength, locomotor activity, and motor coordination. However, these results also show that the expression of Mecp2 is required for motor memory formation, as has been demonstrated for spatial learning in this mouse model [50].

To investigate further how EE improves locomotor activity and motor function in *Mecp2^-/y^* mice, we evaluated the phenotype of the motor cortex to determine whether EE exposure might modulate neuromotor progression by changes in neuronal cytoarchitecture, as has been demonstrated in other rodent models [63]. We evaluated the M1–M2 motor cortex cytoarchitecture at 3 and 7 weeks of age, in WT and *Mecp2^-/y^* mice exposed to either SC or EE. At 3 weeks of age, the RTT phenotype is just starting to appear, whereas at 7 weeks of age, *Mecp2^-/y^* mice showed severe neurological RTT-like symptoms [16]. This was also the time at which we observed motor performance improvement in response to EE exposure. As expected, the motor cortex of 3-week-old *Mecp2^-/y^* mice is not far different from WT. However, at 7 weeks of age, the motor cortex of *Mecp2^-/y^* mice is disorganized, with reduced neuronal complexity and signs of moderate astrogliosis. After 4 weeks of EE exposure, the motor cortex of *Mecp2^-/y^* mice was better organized, had improved neuronal complexity, and had no signs of astrogliosis, seeming similar to WT mice. A more detailed evaluation of pyramidal neurons of cortical layer V was performed, where we measured the dendritic length and the number of dendrites in pyramidal neurons. *Mecp2^-/y^* mice housed in SC have a reduction in the dendritic length and the number of dendrites compared to WT mice. However, these differences were not observed in the pyramidal neurons of *Mecp2^-/y^* exposed to EE. Additionally, second-order dendrites of pyramidal neurons from the motor cortex of *Mecp2^-/y^* mice have less spine density compared to WT, but when exposed to EE, the spine density in *Mecp2^-/y^* mice was both higher than *Mecp2^-/y^* mice in SC and similar to WT. Thus, these results show that early exposure to an EE increases neuronal plasticity and has a positive impact on preventing the damage in motor cortex cytoarchitecture associated with RTT neurological progression. To gain insight into the mechanism by which EE exposure could prevent deterioration of the motor cortex, we next evaluated molecular parameters associated with neurotransmission.

Rett phenotype has been initially associated with a neuronal Mecp2 loss of function [17]. There is growing evidence that Mecp2 deficiency in glia contributes to brain dysfunction and, therefore, to RTT progression [55,56]. Glutamate clearance and production, processes controlled by Glu transporters and Glutamine Synthetase, are abnormal in Mecp2-deficient astrocytes in vitro, probably contributing to the pathological process of RTT [56], and mRNA expression of *Glast* and *Glt-1* glutamate transporters is decreased in *Mecp2*-null astrocytes [56]. Here, we evaluated whether exposure to EE could restore mRNA levels of *Glast* and *Glt-1* to WT levels *in Mecp2^-/y^* mice, and surprisingly, we found no effect of EE exposure on *Glast* nor *Glt1* mRNA expression. Strikingly, we found an increase in Glt-1 protein levels in *Mecp2^-/y^* mice exposed to SC compared to WT mice, and EE exposure decreased Glt-1 protein levels in this mouse model of RTT. Some studies have demonstrated increased Glu levels in the cerebrospinal fluid (CSF) of RTT patients [69,70]. Moreover, Mecp2-deficient microglia release a high level of Glu [55], involving Mecp2 in modulating Glu metabolism. With these antecedents, we did not expect to find increased levels of Glt-1 transporters in *Mecp2^-/y^* mice since this could increase the uptake of Glu, reducing its levels. However, we do not know the localization of these transporters; whether they are in the cell surface is unknown; thus, further experiments are required to answer this and other questions regarding Glu metabolism in *Mecp2^-/y^* mice exposed to EE. Another parameter related to synaptic activity is glutamate receptors. AMPA receptors (AMPAR) subunits are alternatively spliced, generating the “flip” and “flop” variants, which have different kinetic properties [58]. Interestingly, the loss of Mecp2 affects flip/flop splicing of AMPAR genes, leading to a significant splicing shift to the flop inclusion, leading to a faster decay of AMPAR-gated current, and altered synaptic transmission [59], which could impact NMDA receptor activity and compromise synaptic transmission. As expected, we found a significant decrease in the *Gria1* and *Gria2* flip/flop ratio in *Mecp2^-/y^* mice exposed to SC, and according to the behavioral results described above, EE exposure reestablished the flip/flop ratio to WT levels. These results together indicate that EE exposure attenuates the RTT phenotype, and the RTT-like behavior might be mediated by improving neuronal function through reestablishing glutamatergic homeostasis and neurotransmission. However, further analysis must be performed to evaluate electrical synaptic properties.

One of the mechanisms underlying defective neurotransmission is neuroinflammation [71,72]. *Irak1* mRNA expression is upregulated in *Mecp2^-/y^* mice, leading to increased NF-κB signaling [60]. As expected, *Mecp2^-/y^* mice exposed to SC showed an increase in *Irak1* expression, and EE exposure decreased *Irak1* mRNA expression to similar levels as in WT mice. NF-κB signaling is becoming increasingly recognized as a regulator of the growth and morphology of neural processes in the developing and mature nervous system [73]. Therefore, altered NF-κB signaling could play a significant role in RTT progression [60]. Moreover, there is evidence showing that the *Irak1* gene is duplicated in patients with *MECP2* duplication syndrome [74], and that drugs targeting Irak1 in vitro can rescue the inflammatory phenotype associated with RTT [75]. Moreover, the Golgi staining analysis of *Mecp2^-/y^* mice exposed to EE is coherent with the expected phenotype associated with decreased neuroinflammation. Although the effect of EE on Irak1 protein levels was not evaluated, our results shed light on the potential use of EE-based strategies to attenuate *Irak1* expression as a novel non-pharmacological therapeutic option to modulate NF-κB signaling to reduce neuroinflammation in RTT patients. 

Respiratory impairment is one of the leading premature causes of death in RTT patients [7,9]. In RTT mouse models, irregular breathing and hard respiration have been reported [16,17]. The phrenic–diaphragm neuromuscular junction in *Mecp2^-/y^* mice had an increased endplate compared with their WT littermates, and we found that this increase was prevented by exposure to EE in *Mecp2^-/y^* mice. Thus, exposure to a neuronal plasticity-dependent paradigm induces changes in the neuromuscular junction that could underlie the improved neurological and motor phenotype, as well as the increased lifespan observed in response to this experimental paradigm. This improved neuromuscular junction induced by EE exposure could extend to skeletal muscles, improving motor function and locomotor activity associated with EE in *Mecp2^-/y^* mice, and potentially increasing the release of neurotrophic myokines that may be involved in attenuating the progression of the RTT phenotype in this mouse model. As an approach to test this hypothesis, we evaluated the effect of treatment with plasma from young mice with regular motor activity on the progression of the RTT phenotype.

It has been previously demonstrated that plasma treatment from young to aged mice increases neuronal plasticity [49], probably by delivering increased levels of neurotrophic factors like BDNF [62], whose circulating levels are decreased in both RTT patients and *Mecp2^-/y^* mice. Therefore, plasma from young WT mice with regular physical activity could replicate some of the beneficial effects observed in mice exposed to EE. After 2.5 weeks of young plasma IP injections, *Mecp2^-/y^* mice had attenuated the progression of motor and neurological RTT-like symptoms, as evaluated by the ledge test and hindlimb clasping, and showed an increased lifespan. However, IP injections of young plasma did not prevent open arms preference in the elevated plus maze observed in *Mecp2^-/y^* mice. Still, it did improve poor motor coordination in *Mecp2^-/y^* mice, as the time of first arrival and the number of falls from the elevated dowel test were similar to WT mice. Young plasma injections almost reestablished the thickness of the corpus callosum and motor cortex in *Mecp2^-/y^* mice to that in WT mice, preventing the diminished brain architecture observed in *Mecp2^-/y^* mice in SC. The moderate astrogliosis presented in the motor cortex of *Mecp2^-/y^* mice was attenuated by IP plasma injection, and the neuronal cytoarchitecture of pyramidal neurons, as evaluated by dendritic length and dendritic arborization complexity, was re-established by plasma treatment. Hence, our results show the potential therapeutic effect of plasma treatment in attenuating the RTT phenotype and that peripheral signals can reactivate dormant neurodevelopment in RTT.

Currently, there is no cure for RTT syndrome. Several therapeutic avenues have been explored, with the most recent being trofinetide, an FDA-approved drug. Despite its therapeutic potential, adverse side effects such as diarrhea, seizures, and vomiting have been reported [76]. Therefore, finding alternative treatments with few to no adverse side effects, like those presented here, is crucial for improving the quality of life and symptoms of RTT patients.

## 5. Conclusions

Overall, these findings suggest that neuronal plasticity-dependent paradigms, such as enriched environment and plasma transference, attenuate neurological progression associated with Rett syndrome in a murine model of the pathology. These results, with appropriate adaptation and further studies, could serve as the design for non-pharmacological strategies to improve neuronal function, cytoarchitecture, neuromuscular junctions, and inflammation—functional and morphological characteristics that are altered in Rett syndrome and other neurodevelopmental disorders. Hence, these approaches may be used to treat RTT syndrome and other pathologies that share these alterations.

## Figures and Tables

**Table 1:** Primers used for RT-qPCR.

Target Name	Forward Primer	Reverse Primer
*Glast*	ACGGTCACTGCTGTCATT	TGTGACGAGACTGGAGATGA
*Glt* *-1*	CTGGTGCAAGCCTGTTTCC	GCCTGTTCACCCATCTTCC
*Irak1*	ACTACATATGCTGTGAAGAGA	CTCATCCAGAAGCACGTTAGA
*Gria1 flip*	ACACCATGAAAGTGGGAGGTAACT	ACTGGTCTTGTCCTTACTTCCGGA
*Gria1 flop*	GTCCGCCCTGAGAAATCCA	GCACTCGCCCTTGTCGTA
*Gria2 flip*	ACACCATGAAAGTGGGCGGCAACC	ACTGGTCTTTTCCTTACTTCCCGA
*Gria2 flop*	ACACCATGAAAGTGGGCGGCAACC	ACTGGTCTTTTCCTTGGAATCACC
*Gria3 flip*	ATACGATGAAAGTTGGTGGAAATC	ACTGGTCTTGTCCTTACTCCCGGA
*Gria3 flop*	ATACGATGAAAGTTGGTGGAAATC	ACTGGTCTTGTCCTTGGAGTCACC
*Cyc*	GGCAATGCTGGACCAAACACAA	GTAAAATGCCCGCAAGTCAAAAG

## Data Availability

The raw data supporting the conclusions of this article will be made available by the authors upon request.

## Supplementary Materials

The following supporting information can be downloaded at: https://www.mdpi.com/article/10.3390/biom15050748/s1, Figure S1: Differentially expressed genes between *Mecp2^-/y^* and wild-type mice are enriched in genes belonging to immune system processes and pathways. The list of differentially expressed genes between *Mecp2^-/y^* and wild-type mice (see Supplementary File S1) was used as input for the ClueGO app in Cytoscape for Gene Ontology Enrichment Analysis, using the GO ImmuneSystemProcess-EBI-UniProt-GOA-ACAP-ARAP_25.05.2022_00h00: 3113 as the reference database. Default parameters were used to create the ClueGO network. The node size represents the significance (small node represents *p* > 0.1, medium node represented 0.05 > *p* > 0.0005, and big node represented *p* < 0.0005). The different colors on each node represent the specificity of the biological role of the genes. The enriched terms include antigen processing and presentation of peptide antigen via MHC class Ib, positive regulation of pattern recognition receptor signaling pathway, Toll-like receptor 4 signaling pathway, and response to type I interferon.
