# Supplementary figures and images for: Neuronal Plasticity-Dependent Paradigm and Young Plasma Treatment Prevent Synaptic and Motor Deficit in a Rett Syndrome Mouse Model
Source: Biomolecules. 2025 May 21;15(5):748. doi: 10.3390/biom15050748 (PMC12109941; doi:10.3390/biom15050748)

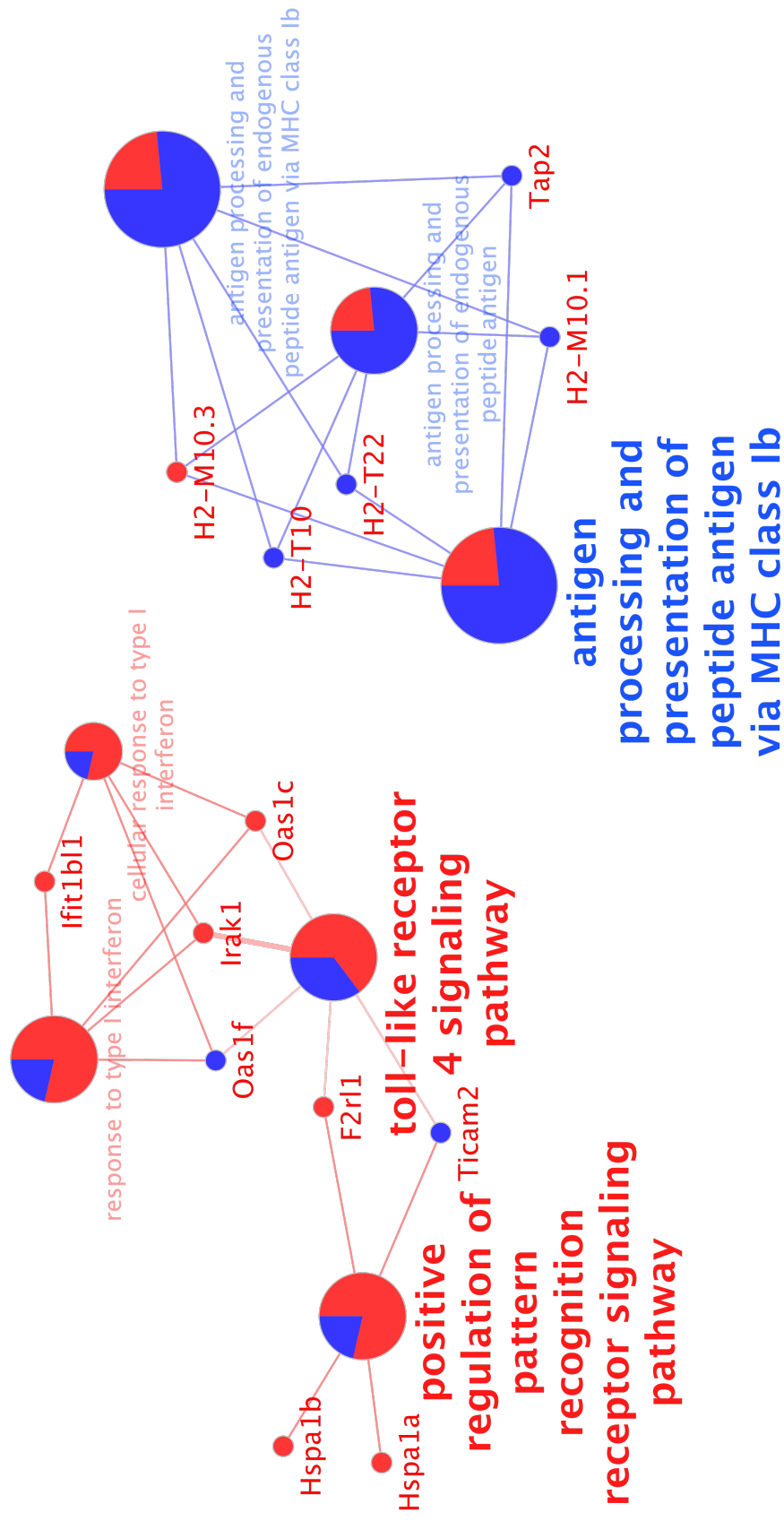

Supplement: Supplementary file 1 [file biomolecules-15-00748-s001.zip › Figure S1.pdf]

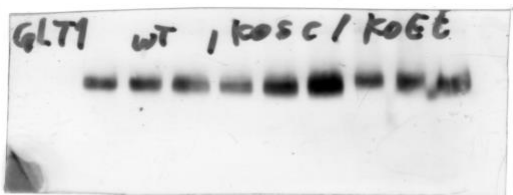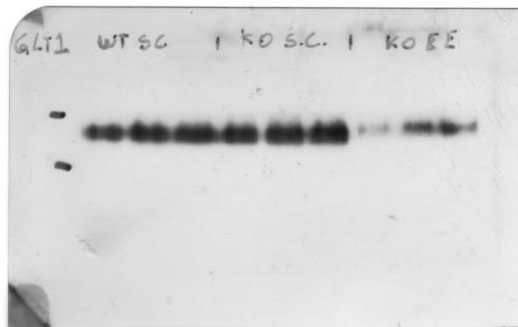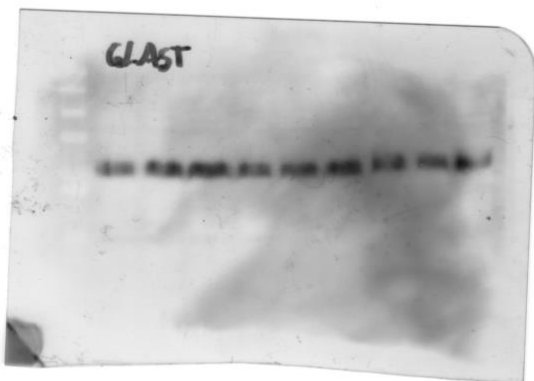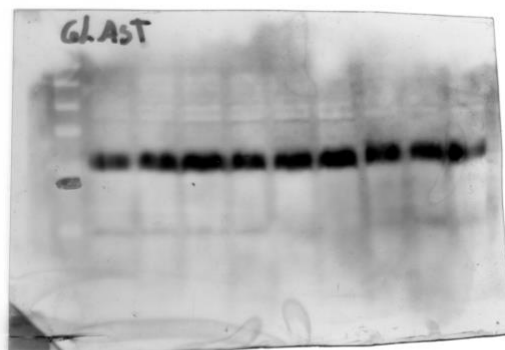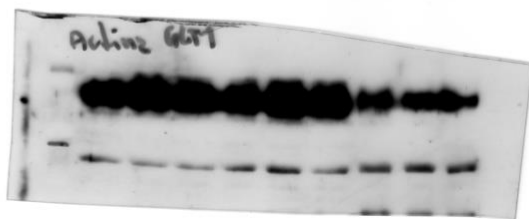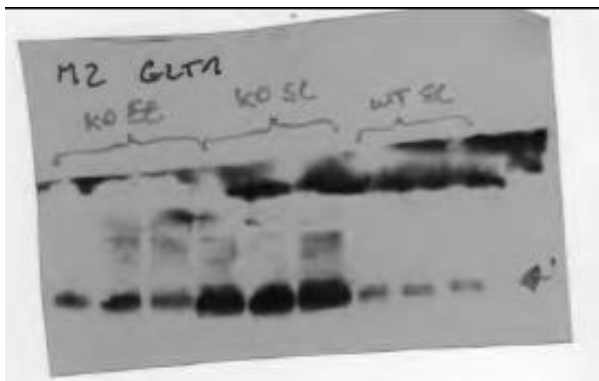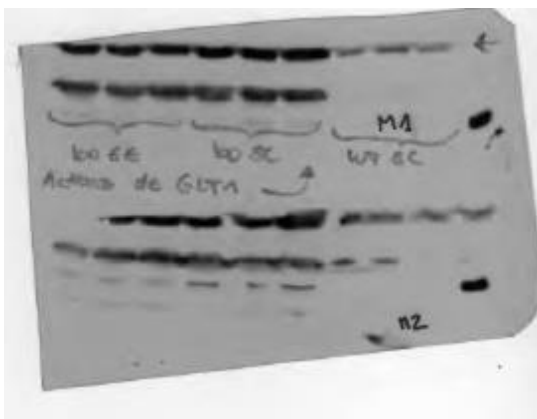

Supplement: Supplementary file 1 [file biomolecules-15-00748-s001.zip › Original Blots.pdf]
